# Journey tracker: driver alerting system with a deep learning approach

**DOI:** 10.3389/frobt.2024.1433795

**Published:** 2024-10-04

**Authors:** N. L. Yashaswini, Vanishri Arun, B. M. Shashikala, Shyla Raj, H. Y. Vani, Francesco Flammini

**Affiliations:** ^1^ Department of Information Science and Engineering, JSS Science and Technology University, Mysuru, Karnataka, India; ^2^ Department of Computer Applications, JSS Science and Technology University, Mysuru, Karnataka, India; ^3^ Department of Innovative Technologies and IDSIA—Dalle Molle Institute for Artificial Intelligence, University of Applied Sciences and Arts of Southern Switzerland, Lugano, Switzerland

**Keywords:** media research lab, swish activation function, baseline behavior, custom EfficientNet, pupil detection

## Abstract

Negligence of public transport drivers due to drowsiness poses risks not only to their own lives but also to the lives of passengers. The designed journey tracker system alerts the drivers and activates potential penalties. A custom EfficientNet model architecture, based on EfficientNet design principles, is built and trained using the Media Research Lab (MRL) eye dataset. Reflections in frames are filtered out to ensure accurate detections. A 10 min initial period is utilized to understand the driver’s baseline behavior, enhancing the reliability of drowsiness detections. Input from drivers is considered to determine the frame rate for more precise real-time monitoring. Only the eye regions of individual drivers are captured to maintain privacy and ethical standards, fostering driver comfort. Hyperparameter tuning and testing of different activation functions during model training aim to strike a balance between model complexity, performance and computational cost. Obtained an accuracy rate of 95% and results demonstrate that the “swish” activation function outperforms ReLU, sigmoid and tanh activation functions in extracting hierarchical features. Additionally, models trained from scratch exhibit superior performance compared to pretrained models. This system promotes safer public transportation and enhances professionalism by monitoring driver alertness. The system detects closed eyes and performs a cross-reference using personalization data and pupil detection to trigger appropriate alerts and impose penalties.

## 1 Introduction

In the realm of public transportation, countless individuals rely on drivers daily to reach their destinations safely. This necessitates a mechanism that can strictly monitor driver performance and alert them whenever drowsy driving is detected. Existing solutions use physiological, behavioural, and vehicle-based methods to detect drowsiness. A combination of these methods, known as hybrid methods, is emerging as a superior approach. Physiological measures involve using ECG sensors to track variations in ECG signal frequency and EEG sensors to monitor brain activity through EEG signals. Behavioural measures detect drowsiness based on driver behaviour, such as eye closures, yawning, and head tilting. Vehicle-based measures use lane deviation to detect drowsiness. Current challenges with these methods include late detection, where even a fraction of a second of negligence can lead to an accident. Lane detection mechanisms that alert drivers only after the vehicle has deviated from its lane are often too late. Additionally, the need for sensors to be physically placed on the driver is a setback for physiological measures. Behavioural-based measures, which use extensively trained deep learning models to detect drowsy behaviour, face challenges of high computational cost and complexity, leading to delayed system performance and unreliability in critical situations. Moreover, increased false alerts from existing systems can distract drivers.

To address these challenges, the Journey Tracker System has been developed, employing an advanced model architecture inspired by Efficient Net principles for closed eye and open eye detection. This design balances model complexity, computational cost and performance ensuring accurate and timely alerts for drivers. The system also features sophisticated reflection detection and filtering techniques, which remove frame reflections to enhance alert accuracy. Personalization and pupil detection further refine the system by cross-referencing detected closed eyes with individual pupil data, significantly reducing false alerts. By combining efficient model architecture, advanced filtering and personalized monitoring, the Journey Tracker System provides precise and reliable drowsiness detection, delivering alerts exactly when needed and significantly improving driver safety.

## 2 Related work

Research on drowsy driver detection offers a promising avenue to address this public health concern, potentially leading to safer roads and improved wellbeing for all. Driver drowsiness detection is a crucial aspect of ensuring road safety, and various methods have been proposed to address this issue. One approach involves monitoring eye aspect ratios (EAR) and facial expressions using the iBUG-300w dataset. This method achieved 80% accuracy in eye detection and 78% in drowsiness detection. However, future work should focus on addressing challenges related to obstructions and lighting variations for real-time monitoring (Saranya et al., 2023). Another approach utilizes computer vision techniques and a Raspberry Pi to detect driver fatigue and yawning in real-time, achieving 73.74% accuracy. Potential improvements are necessary for real-time monitoring, especially under low-light conditions (Dehankar et al., 2023). A method focusing on face and eye region detection to determine drowsiness using an eye aspect ratio threshold achieved 97% accuracy. Future work should improve performance under varying lighting conditions and address frame reflections (Kannan et al., 2023).

Facial expression analysis using Open Face software and Decision Trees achieved 91% accuracy. The system’s effectiveness in nighttime driving remains an area for improvement (Abad et al., 2021). Multimodal representations using K-NN and SVM learn from driving videos, physiological signals, and steering wheel movements. Future work should address data scarcity and involve diverse participants to improve accuracy (Qian et al., 2022). A CNN on Raspberry Pi, using the Viola-Jones algorithm, trained to detect closed eyes, open eyes, yawning and non-yawning achieved 80% accuracy. Addressing nighttime driving performance issues could enhance practical utility (S. I et al., 2022). Driver’s facial landmark detection and the 2s-STGCN model achieved 93.4% accuracy on the YawDD dataset and 92.7% on the NTHU-DDD dataset. Future efforts should focus on robust methods against illumination changes and occlusions (Bai et al., 2022). Eye location detection using Haar Cascade and EAR provides another method. Ensuring reliable performance under various conditions remains a challenge (Prasath et al., 2022). EEG signals recorded with commercial headsets provide data for detecting drowsiness, achieving high accuracy. Vehicle-based measures offer non-intrusive data collection but face external disturbances (Rezaee et al., 2022). The study using PPG signals to detect heartbeat peaks achieved a 96% success rate in detecting drowsiness. Sensor connection issues present challenges (Purnamasari & Hazmi, 2018). Physiological monitoring allows earlier drowsiness prediction, while vehicle-based methods detect drowsiness (Perkins et al., 2023). Self-supervised learning methods enable training with unlabelled datasets, demonstrating great potential for drowsiness detection applications (Mou et al., 2023). An embedded system using a Raspberry Pi, RGB camera, and cloud computing for real-time drowsiness detection includes a dataset with diverse samples. Addressing performance under various driving conditions remains essential (Khan et al., 2023).

However, one critical aspect that emerges from these studies is the necessity for the system to familiarize itself with the individual driver’s behaviour over a period of approximately 10. By studying the real-time behaviour of the driver initially, the system can better discern the signs of drowsiness and effectively alert the driver when necessary.

To further enhance the effectiveness of drowsy driver detection, several pertinent questions arise, guiding the direction of this research: How can a model architecture designed by specifying its own structure and layers, using the design principles of EfficientNet perform better compared to a pretrained EfficientNet model in terms of accuracy and inference speed ^[1]^? How can the journey tracker system effectively deal with reflections on the frames, which hinder the system’s performance ^[2]^? Can incorporating input received directly from the driver help to improve the real-time monitoring of the driver’s alertness ^[3]^? How does the information gathered during the 10 min personalization phase contribute to understanding the driver’s behaviour and help in reducing false alerts ^[4]^? and finally, how can the system ensure that drivers feel comfortable with their frames constantly being captured, while also addressing privacy and ethical considerations ^[5]^? These questions aim to provide insights and solutions to enhance driver alertness and road safety.

## 3 Methodology

In the methodology section, the strategies to address the research questions outlined in the related work section are detailed.

### 3.1 Functional overview

**FIGURE 1:**
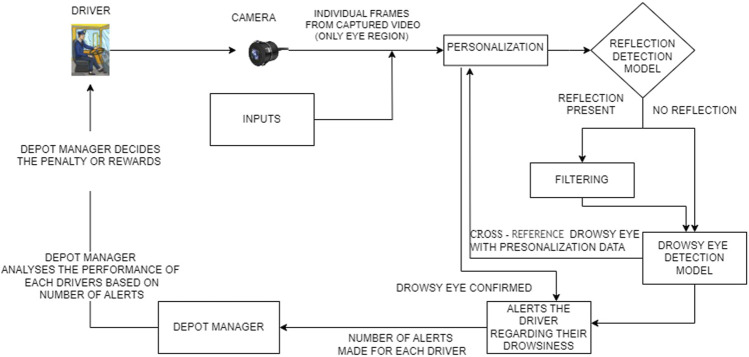
Functional overview.

As depicted in Figure 1, the system captures video frames of the driver’s eyes using a camera. Driver inputs are initially used to determine the optimal frame capture rate. A 10 min personalization phase follows, where the system establishes a baseline of the driver’s eye behavior. Then, during the monitoring phase, the system continuously analyzes captured frames for signs of drowsiness. To ensure accuracy, both personalization and monitoring phases involve reflection detection and filtering. Additionally, the system classifies the driver’s eyes as open or closed in each frame. By cross-referencing this information with the personalization data and pupil detection, the system determines drowsiness and issues voice alerts to warn the driver if necessary.

### 3.2 Architectural design of the proposed model and training strategy comparison

#### 3.2.1 Design principles of EfficientNet

This architecture ensures superior performance with fewer parameters and computations compared to other popular architectures like ResNet and InceptionV3. ResNet 50, while addressing the vanishing gradient problem with residual connections, introduces significant complexity and computational overhead, making it less suitable for real-time applications in resource-constrained environments. InceptionV3, although highly effective for tasks like object identification due to its use of multiple convolutions in parallel, is less suited for drowsiness detection. This task requires understanding the subtle differences between closed and open eyes through hierarchical feature extraction, which goes beyond mere object identification. EfficientNet with its balanced scaling approach, ensures efficiency as model size increases, allowing it to perform efficiently across different model sizes without sacrificing performance. This makes it ideal for the nuanced task of detecting drowsiness in drivers. Comparative metrics highlight EfficientNet’s advantages over InceptionV3 and ResNet 50, demonstrating its superior accuracy, precision and recall rates, as detailed in Table 1. These factors collectively justify the choice of EfficientNet for a robust, efficient solution that excels in performance, scalability and practical deployment for real-time drowsiness detection and alerting systems.

**TABLE 1:** Comparison of pretrained and custom model performance metrics.

	Efficientnet b0 model	Inception v3	Resnet 50
Test accuracy	90.35%	92.85%	87.06%
Test loss	0.3594	0.1602	0.2223
Validation accuracy	**98.81%**	98.18%	88.43%
Validation loss	0.0351	0.0502	0.0500
Precision Closed Open	**0.83** **1.00**	0.88 0.98	0.80 0.98
Recall Closed Open	**1.00** **0.81**	0.98 0.88	0.98 0.77

Bold values indicate the best results in the comparison presented in the Table.

#### 3.2.2 Custom EfficientNet model architecture vs. EfficientNet pretrained models

The customized EfficientNet architecture for drowsiness detection is tailored to improve performance and efficiency compared to the original EfficientNet B0. Notably, the number of blocks has been reduced from 7 in the original to just 4 in the customized version, streamlining the model for faster inference. The convolutional layers have been modified with specific adjustments in filter sizes and strides—for example, the customized version uses 3 × 3 and 5 × 5 filters with strides varying from 1 to 2, focusing on capturing subtle patterns between open and closed eyes. This contrasts with the original EfficientNet B0, which uses a broader range of MBConv layers optimized for a wide variety of image features. Additionally, the output layer has been simplified to suit binary classification, reducing the number of neurons and computational overhead. While the original model is pretrained on the ImageNet dataset with 1,000 classes, the customized version is trained on the MRL Eye Dataset, which is specifically curated for eye state detection, significantly enhancing its accuracy in this application. Moreover, dropout rates have been adjusted to balance model complexity and prevent overfitting, ensuring robust performance in real-time scenarios, particularly in resource-constrained environments.

**FIGURE 2:**
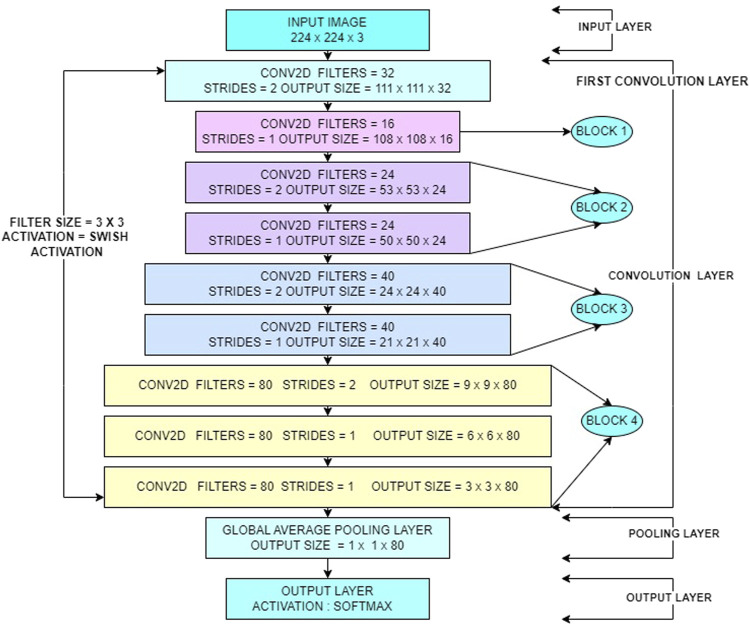
Model architecture diagram.

By carefully selecting architectural parameters, custom EfficientNet models can achieve comparable or superior performance with fewer parameters, reducing memory footprint and inference time. This assertion is supported by Table 2, which provides a comprehensive comparison between the performance of the custom EfficientNet model and pretrained EfficientNet models. It highlights key metrics such as accuracy, inference speed and memory usage, demonstrating the efficiency of the custom EfficientNet model architecture in addressing the specific requirements of the task at hand. Figure 2 provides the architecture diagram of the custom EfficientNet model. With this the first research question is addressed ^[1]^.

**TABLE 2:** Comparison of pretrained EfficientNet B0 and custom model metrics.

	EfficientNet B0 model	Custom EfficientNet model
Test accuracy	90.35%	**94.66%**
Test loss	0.3594	0.1366
Validation accuracy	98.81%	98.71%
Validation loss	0.0351	0.0404
Precision	91.93%	95.17%
Recall	90.35%	94.66%
Model size	48.30 mb	2.26 mb

Bold values indicate the best results in the comparison presented in the Table.

#### 3.2.3 Architectural design of custom EfficientNet model

Custom EfficientNet model prioritizes balanced width and depth for efficient feature extraction. The core building block, the efficientnet_block function, processes data through a fixed number of convolution layers with Batch Normalization and Swish Activation.

The efficientnet_model function stacks these blocks in a predetermined order, progressively increasing filter sizes within each block to capture finer details. This balanced approach between expanding filter dimensions (width) and stacking more blocks (depth) ensures efficient feature learning without sacrificing accuracy. Finally, the model incorporates Global Average Pooling, Dropout and regularization to avoid overfitting and a Dense layer with SoftMax activation for classification. Compiled with an optimizer, loss function and training callbacks this architecture achieves drowsiness detection through efficient feature extraction and balanced model complexity.

Output from the average pooling layer will be of size 1x1x80 which is flattened to vector of dimension 80x1 .

**Logits** are calculated by taking the dot product of the output of a fully connected layer and the weights followed by adding a bias term.

$${\text{Logit}}_{0}\left(z_{0}\right)=\sum_{i=0}^{80}⬚\;\text{Output}\left[i\right]\;x\;w_{1}$$ (1)

$${\text{Logit}}_{1}\left(z_{1}\right)=\sum_{i=0}^{80}⬚\;\text{Output}\left[i\right]\;x\;w_{2}$$ (2)

where, the value of each element in the flattened vector of dimension 80x1 is represented by $Output\left[\mathrm{i}\right]$ and w1 and w2 represent the weights associated with each class “closed” and “open” respectively.

**SoftMax activation function** is applied to the Logits to obtain the class probabilities. The probability of class 0 (P_0_) and class 1 (P_1_) are calculated using the SoftMax function.

$$P_{0}=\frac{e^{z_{0}}}{e^{z_{0}}+e^{z_{1}}}$$ (3)

$$P_{1}=\frac{e^{z_{1}}}{e^{z_{0}}+e^{z_{1}}}$$ (4)

where, $z_{0}$ and $z_{1}$ represent Logits obtained from Equations 1, 2. *e* represents Euler’s number, which is a mathematical constant approximately equal to 2.71828.

**Cross-entropy loss function** is used to measure the difference between the predicted probabilities and the true label. y_i_ is the true label (1 for the correct class, 0 for the incorrect class. P_i_ is the predicted probability of class i, obtained using the SoftMax function in Equations 3, 4.

$$L=-\sum_{i=0}^{1}y_{i}\log\left(P_{i}\right)$$ (5)

**Regularization** terms are added to the loss function to penalize large weights and prevent overfitting.

$$R=\frac{\lambda }{2}\left({\left\|w_{1}\right\|}^{2}+{\left\|w_{2}\right\|}^{2}\right)$$ (6)

where λ represents the regularization parameter.

**Total loss** or the sum of the cross-entropy loss and regularization is calculated as in Equation 7.

Total Loss=L+R (7)

where L and R represent the Loss Function value obtained from Equation 5. R represents the regularization term obtained from Equation 6.

The update rule for the weights using the gradient descent with **Learning Rate** is as in Equation 8.

$$w_{\text{new}}=w_{\text{old}}-\alpha \times \frac{\partial L}{\partial \omega }$$ (8)

where $w_{old}$ represents the old weight parameter value. $w_{new}$ represents the new updated weight parameter value. ∝ represents the Learning Rate. ∂L/∂ω represents the gradient of the loss function with respect to weight.

**Momentum** update rule for the weight W is as in Equation 9.

$$w_{\text{new}}=w_{\text{old}}-\alpha \times v_{t+1}$$ (9)

$$v_{t+1}=\beta \times v_{1}+\left(1-\beta \right)\times \frac{\partial L}{\partial \omega }$$ (10)

$v_{t}$ represents the momentum at time *t*. $v_{t+1}$ represents the updated value of the momentum term after some time interval as obtained in Equation 10. β represents the momentum parameter.

## 4 Experimental setup

### 4.1 Dealing with reflections

The VGG16 architecture is employed for the reflection detection model. Although resource-intensive with larger datasets, its exceptional feature extraction capabilities proved beneficial for our smaller dataset. Real-time captured frames are initially processed through VGG16 for reflection identification. If reflections are detected, Color normalization is applied to these frames. Color normalization is crucial for removing reflections and enhancing image quality. This process involves converting the image from the RGB color space to the LAB color space, where the LAB model separates lightness (L) from color information (A and B). Contrast Limited Adaptive Histogram Equalization (CLAHE) is then applied to the L channel to enhance contrast while preserving color of the frame. LAB color space facilitates device-independent color representation and allows for independent manipulation of lightness and color components. Overall, color normalization utilizing LAB color space and CLAHE improves image quality, enhances details and ensures natural color appearance. With this normalization the reflections in the frames are removed which was a hindrance to the closed eye, open eye detection model to have accurate detections. The normalized frames are then passed as inputs to the subsequent EfficientNet model for closed and open eye detection. This approach underscores the importance of combining multiple models to achieve superior performance in complex tasks. With this the second research question is addressed [2].

### 4.2 Inputs from the driver

Driver inputs play a critical role in assessing their level of restlessness and potential risk of fatigue-related issues while driving. Three essential variables are collected: previous sleep duration, previous working hours and any medication taken. These inputs are fundamental in determining whether a driver may be experiencing restlessness due to extended working hours or insufficient sleep. Based on this information, the system adjusts its monitoring levels accordingly. If a driver report extended working hours or inadequate sleep, indicating potential restlessness the system intensifies monitoring by increasing the frequency of eye frame analysis for detecting drowsiness. This proactive approach aims to mitigate the heightened risk of driver drowsiness and ensures timely intervention to maintain safety standards. Conversely, when a driver reports adequate rest and arrives alert, the system adjusts the frame analysis rate to allow longer intervals between checks for drowsiness. This adaptive strategy optimizes system resources while ensuring continuous monitoring aligned with the driver’s condition. By monitoring these key inputs, including sleep duration, working hours and medication effects the system gains valuable insights into the driver’s state and effectively manages fatigue-related risks through targeted adjustments in monitoring intensity. This approach enhances the practicality of the system by enabling strict monitoring to provide accurate alerts in critical situations, thereby ensuring proactive measures are taken to uphold safety standards during driving operations. With this the third research question is addressed ^[3]^.

### 4.3 Cross-referencing drowsy eye detection

The drowsy eye detected frames in the monitoring phase shall be cross referred using personalization data and pupil detection method before alerting the driver to reduce false alerts and have more reliable system.

#### 4.3.1 Personalization data

The initial 10 min phase for personalization is chosen because drivers typically do not experience drowsiness immediately upon starting to drive. In most cases, they remain awake and alert for at least 10 min. This initial period provides an opportunity to establish the driver’s baseline behaviour, which is crucial for accurate monitoring and to prevent false alerts. Throughout this phase, thresholds for open and closed eye blink rates are determined by dividing the 10 min into multiple 10 s segments and recording detections. Maximum counts for open and closed eyes across these segments are set as thresholds. During ongoing monitoring, if a closed eye is detected, the system examines the last 10 s window of detections. If the count exceeds the threshold, it confirms a closed eye condition and triggers alerts. Similarly, detecting an open eye with a count twice the threshold indicates potential drowsiness with open eyes, prompting necessary actions. This method ensures validation of closed eye detections from the deep learning model, enhancing alert accuracy and minimizing false alert.

#### 4.3.2 Pupil detection

**FIGURE 3:**
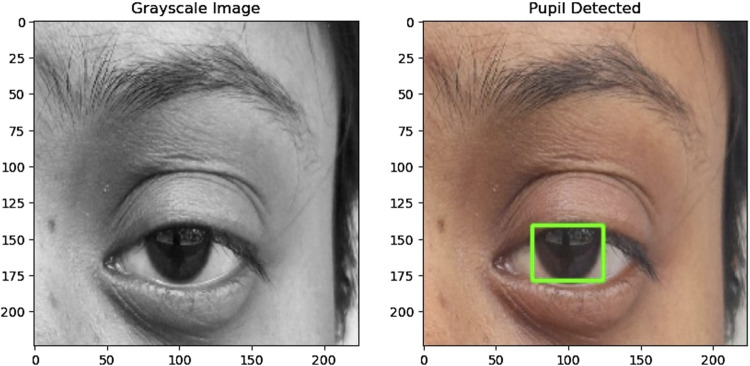
(Left) Grayscale image of the eye used for detection. (Right) The same eye image with a bounding box drawn around the detected pupil region, demonstrating the accuracy and effectiveness of the pupil detection algorithm.

**FIGURE 4:**
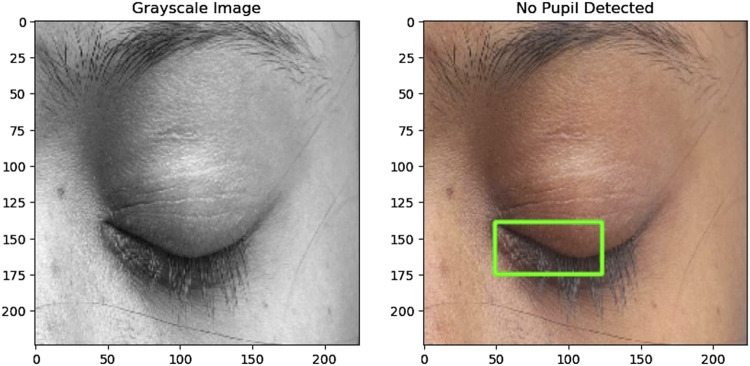
(Left) Grayscale image of the eye used for detection. (Right) The same eye image with no bounding box, indicating no pupil was detected in this instance.

The preprocessing of the eye region for pupil detection involves several steps aimed at preparing the image for accurate analysis. Initially, the eye region is converted to grayscale and thresholded to create a binary image emphasizing the pupil where the pixel values below 75 are converted to pixel value 0 representing black and then pixel values above 75 are converted to 255 representing white. Morphological closing is then applied to smooth irregularities and enhance the pupil’s boundaries. Contours are detected in the processed image, filtering out noise and small artifacts to focus on significant features. The largest contour is identified as the pupil, and its bounding rectangle is calculated to determine its position and size. If a valid pupil is detected, a green rectangle is drawn around it on the original eye region image. This process facilitates the accurate detection of the pupil, enabling further analysis and tracking of eye movements. The function returns True if a pupil is detected and False otherwise, indicating the success of the detection process. Figures 3, 4 illustrate how pupil detection works, showing a grayscale image and a bounding box around the detected pupil region. With this, the fourth research question is addressed ^[4]^.

### 4.4 Privacy consideration

During personalization and real time monitoring phase only the eye frames are captured to address privacy concerns and ethical considerations. By focusing solely on the eyes, sensitive facial features are not recorded, respecting the privacy of the driver. This approach ensures that only the necessary data for eye detection is collected, minimizing any potential intrusion into the driver’s privacy. With this the fifth research question is addressed ^[5]^.

## 5 Results

To ensure balanced training, the drowsiness detection dataset was split to contain 40,837 images for each class (open and closed eyes). These images were divided for training (32,670 images/class), validation (8,168 images/class) and testing (1,612 images/class). To assess model performance, metrics like test accuracy, test loss, validation accuracy, validation loss, precision and recall were used. Initially, these metrics were used to compare the performance of pre-trained models (InceptionV3, ResNet 50, EfficientNet B0), with EfficientNet B0 demonstrating the best performance with 98.81% of validation accuracy as shown in Table 1. Subsequently, the focus shifted to EfficientNet B0. The same metrics were employed to evaluate the performance difference between the pre-trained EfficientNet B0 and a custom model built using EfficientNet design principles. Results shown in Table 2 proved that custom model with EfficientNet Design principles performed better compared to pretrained EfficientNet B0 being more precise. Furthermore, the custom EfficientNet model underwent fine-tuning with various activations and combinations of learning and regularization pairs. It was discovered that Swish activation with learning rates of 0.001 and 0.01 yielded better results in terms of precision and recall, as demonstrated in Tables 2–4.

**TABLE 3:** Comparison of the performance of custom model trained with different activations in the convolutional layers.

	ReLU	Sigmoid	Tanh	Swish
Precision				
Closed	0.94	0.83	0.92	0.90
Open	0.92	0.95	0.82	1.00
Recall				
Closed	0.91	0.96	0.78	1.00
Open	0.95	0.81	0.94	0.90
Accuracy	93.14%	88.33%	86.14%	**94.66%**

Bold values indicate the best results in the comparison presented in the Table.

**TABLE 4:** Performance comparison of custom model trained with different learning and regularization rates with swish as activation function.

Learning rate and regularization rate pairs Swish activation function	(0.001, 0.01)	(0.01, 0.001)	(0.001,0.001)
Precision			
Closed	0.90	0.96	0.94
Open	1.00	0.91	0.90
Recall			
Closed	1.00	0.90	0.89
Open	0.90	0.97	0.95
Accuracy	**94.6%**	93.3%	91.7%

Bold values indicate the best results in the comparison presented in the Table.

### 5.1 Swish activation: a smooth performer

The choice of activation function plays a crucial role in a neural network’s performance. This model employs the Swish activation function, a recent addition known for its benefits over traditional options like ReLU, sigmoid, and tanh. Unlike ReLU, which sets negative values to zero, Swish offers a smooth, continuous transition from negative to positive values. This smoothness in the activation function translates to improved optimization during the training process, as evidenced by the results presented in Table 3.

### 5.2 Using SoftMax activation with categorical cross-entropy in binary classification

Through extensive experimentation, observed that the combination of SoftMax activation and categorical cross-entropy yielded significantly better performance metrics compared to using sigmoid activation and binary cross-entropy. Specifically, our model achieved a validation accuracy of 98.71% and a test accuracy of 94.66% with SoftMax, compared to a validation accuracy of 50% and a test accuracy of 69% with sigmoid.

#### 5.2.1 Normalization effect

The SoftMax activation function normalizes the outputs into a probability distribution, where the sum of all probabilities equals 1. This normalization helps in cases where the decision boundary between classes (open and closed eyes) is not sharp, providing more stable and reliable gradients during training.

The mathematical expressions for the model performance metrics are as follows:

$$\text{Accuracy}\left(A\right):\;A=\frac{TP+TN}{TP+TN+FP+FN}$$

$$\text{Precision}\left(P\right):\;P=\frac{TP}{TP+FP}$$

$$\text{Recall}\left(R\right):\;R=\frac{TP}{TP+FN}$$

$$F1\;–\text{ Score}\left(F1\right):\;F=2\times \frac{P\times R}{P+R}$$

Where TP is True Positives, TN is True Negatives, FP is False Positives and FN is False Negatives.

## 6 Conclusion

Through meticulous experimentation and analysis, the custom model architecture, incorporating the Swish activation function, a learning rate of 0.001, and a regularization rate of 0.01, significantly enhances the reliability and effectiveness of the journey tracking system for detecting driver drowsiness in real-time. Fine-tuning hyperparameters and evaluating model architectures resulted in a remarkable accuracy rate of 95%, surpassing the performance of pre-trained models. This optimized system ensures the safety of passengers and drivers, promoting professionalism and accountability within the transportation sector. Comprehensive monitoring and personalized alerts, represent a significant advancement in ensuring driver alertness throughout the journey.

## Data Availability

The original contributions presented in the study are included in the article/supplementary material, further inquiries can be directed to the corresponding author.
